# Associations of the Dietary Iron, Copper, and Selenium Level With Metabolic Syndrome: A Meta-Analysis of Observational Studies

**DOI:** 10.3389/fnut.2021.810494

**Published:** 2022-02-01

**Authors:** Jun Ding, Qi Liu, Ze Liu, Hongbin Guo, Jieyu Liang, Yi Zhang

**Affiliations:** ^1^Changsha Social Work College, Changsha, China; ^2^Department of Orthopaedics, Xiangya Hospital, Central South University, Changsha, China; ^3^National Clinical Research Center for Geriatric Disorders, Xiangya Hospital, Central South University, Changsha, China

**Keywords:** dietary iron level, dietary copper level, dietary selenium level, metabolic syndrome, meta-analysis, observational studies

## Abstract

**Background:**

Epidemiological studies have investigated the associations of dietary iron, copper, and selenium level with metabolic syndrome (MetS). However, their results are conflicting. This meta-analysis of observational study was, therefore, employed to investigate the associations above.

**Methods:**

A comprehensive literature search was employed using PubMed, Web of Science, Embase, and Scopus database up to October 2021 (no restriction was set for the initiate time). The pooled relative risk (RR) of MetS for the highest vs. lowest dietary iron, copper, and selenium level was estimated, respectively.

**Results:**

A total of 14 observational studies (55,131 participants) were identified as meeting the inclusion criteria. Specifically, 7 studies were related to the dietary iron level. The overall multivariable adjusted RR demonstrated that the dietary iron level was positively associated with MetS (RR = 1.27, 95% CI: 1.12–1.44; *p* < 0.001). With regard to the dietary copper level, 7 studies were included for meta-analysis. The overall multivariable adjusted RR showed that the dietary copper level was inversely associated with MetS (RR = 0.85, 95% CI: 0.78–0.93; *p* < 0.001). In addition, 4 studies were specified for the dietary selenium level. The overall multivariable adjusted RR indicated that the dietary selenium level was inversely associated with MetS (RR = 0.77, 95% CI: 0.63–0.95; *p* = 0.01) as well.

**Conclusion:**

Our results suggest that the dietary iron level is positively associated with MetS, whereas a negative association between the dietary copper and selenium level and MetS is obtained. Further large well-designed prospective cohort studies are warranted to elaborate on the issues examined in this study.

## Introduction

Elevated waist circumference, blood pressure, fasting blood glucose, triglycerides, and decreased high-density lipoprotein cholesterol (at least three of the five above metabolic abnormalities) are considered as the presence of metabolic syndrome (MetS) (1). MetS is a well-known attributable risk to diabetes, stroke, and coronary heart disease epidemic (2–4). Moreover, MetS increases the incidence of atherosclerotic cardiovascular disorder and complication that decreases longevity (5). However, the subjects suffering from MetS are progressively growing worldwide and the current global prevalence of MetS is between 11.6 and 62.5% (6). Although the etiology of MetS is not well understood yet, dietary factors are considered to be involved in MetS (7–11).

Micronutrients are important factors for cellular and biochemical functions (release of energy for synthesis and movement) (12). Iron, copper, and selenium are considered to be significant micronutrients and their dietary sources are meat, seeds, heme, tea, milk, nuts, cereals, eggs, fish, and so on (13–18). Iron is one of the most abundant elements, which plays a significant role in various cellular processes, such as iron-dependent signaling, cellular respiration, DNA replication and synthesis, nucleic acid repair, and energy metabolism (19–21). Iron consumption, uptake, transfer, and storage are involved to maintain iron homeostasis (22). However, excess iron leads to inflammation and tissue damage, produces hydroxyl radicals (Haber–Weiss–Fenton reactions), which cause oxidative damage to cellular components (lipids, proteins, and DNA) (23, 24). On the contrary, copper, a component of extracellular superoxide dismutase (25), is essential for iron uptake and signaling in eukaryotic organisms, energy metabolism, reactive oxygen species, and detoxification (26). In addition, copper plays an essential role in mitochondrial function and signaling involving mitophagy, bioenergetics, and dynamics, which affect cell fate by metabolic reprogramming (26). Selenium, also an essential micronutrient, is necessary to maintain the different cellular functions, such as signaling transduction pathways and immune-endocrine function (18). Moreover, selenium incorporates into selenoproteins and selenium-dependent enzymes (e.g., glutathione peroxidases), which are involved in intracellular redox regulation and modulation (27). Since oxidative stress and inflammation play a significant role in the pathophysiology of MetS (28), the dietary iron, copper, and selenium level is considered to be closely related to MetS.

A number of observational studies have been employed to investigate the associations of the dietary iron, copper, and selenium level with MetS (29–42). However, their results are still conflicting. Thus, this meta-analysis of observational studies is employed to further investigate the above associations. It is hypothesized that the dietary iron level is positively associated with MetS, whereas the dietary copper and selenium level is inversely associated with MetS.

## Materials and Methods

### Search Strategy

Our meta-analysis was performed according to the Preferred Reporting Items for Systematic Reviews and Meta-analyses (PRISMA) guidelines (43). The PubMed, Web of Science, Embase, and Scopus electronic databases were searched up to October 2021 (no restriction was set for the initiate time) by using a combination of keywords that related to MetS (“metabolic syndrome”), iron (“iron,” “Fe”), copper (“copper,” “Cu”), and selenium (“selenium,” “Se”). No language restriction was set in the search strategy. We screened the titles and abstracts of all the articles and then read the full articles to identify the eligible studies.

### Study Selection

The titles, abstracts, and full texts of all the retrieved studies were reviewed by two researchers independently. Disagreements were resolved by discussions. The included studies were required to meet the following criteria: (1) the study design is an observational study; (2) the outcomes include the associations of the dietary iron, copper, and selenium level with MetS; and (3) the relative risk (RR) or odds ratio (OR) with 95% CI was reported. The exclusion criteria were listed as follows: (1) duplicated or irrelevant articles; (2) reviews, letters, or case reports; (3) randomized controlled trials; and (4) non-human studies.

### Data Extraction

The data were extracted by two researchers independently and disagreements were resolved by discussions. The information about the first author and year of publication, location, age, gender, sample size, study design, adjustments, exposure, category of exposure, effect estimates, and diagnostic criteria of MetS was collected. The corresponding effect estimates with 95% CIs for the highest vs. lowest dietary iron, copper, and selenium level and MetS was extracted (adjusted for the maximum number of confounding variables).

### Quality Assessment

The Newcastle–Ottawa Scale (NOS) criteria for non-randomized studies were employed to assess the quality of each included study. The NOS is based on three broad perspectives: (1) the selection process of the study cohorts; (2) the comparability among the different cohorts; and (3) the identification of exposure or outcome of the study cohorts. Disagreements with respect to the methodological quality were resolved by mutual consultation.

### Statistical Analyses

The RR for MetS was the outcome measure in this meta-analysis. The *I*^2^ statistic, which measures the percentage of total variation across studies due to heterogeneity, was examined (*I*^2^ > 50% was considered as heterogeneity). If significant heterogeneity was observed among the studies, a random-effects model was used; otherwise, a fixed-effects model was accepted. Begg's test was employed to assess the publication bias (44). A *p*-value of < 0.05 was considered statistically significant. Moreover, subgroup analysis for study design, diagnostic criteria of MetS, sample size, exposure assessment, type of iron, and the population was employed.

## Results

### Study Identification and Selection

**Figure 1:**
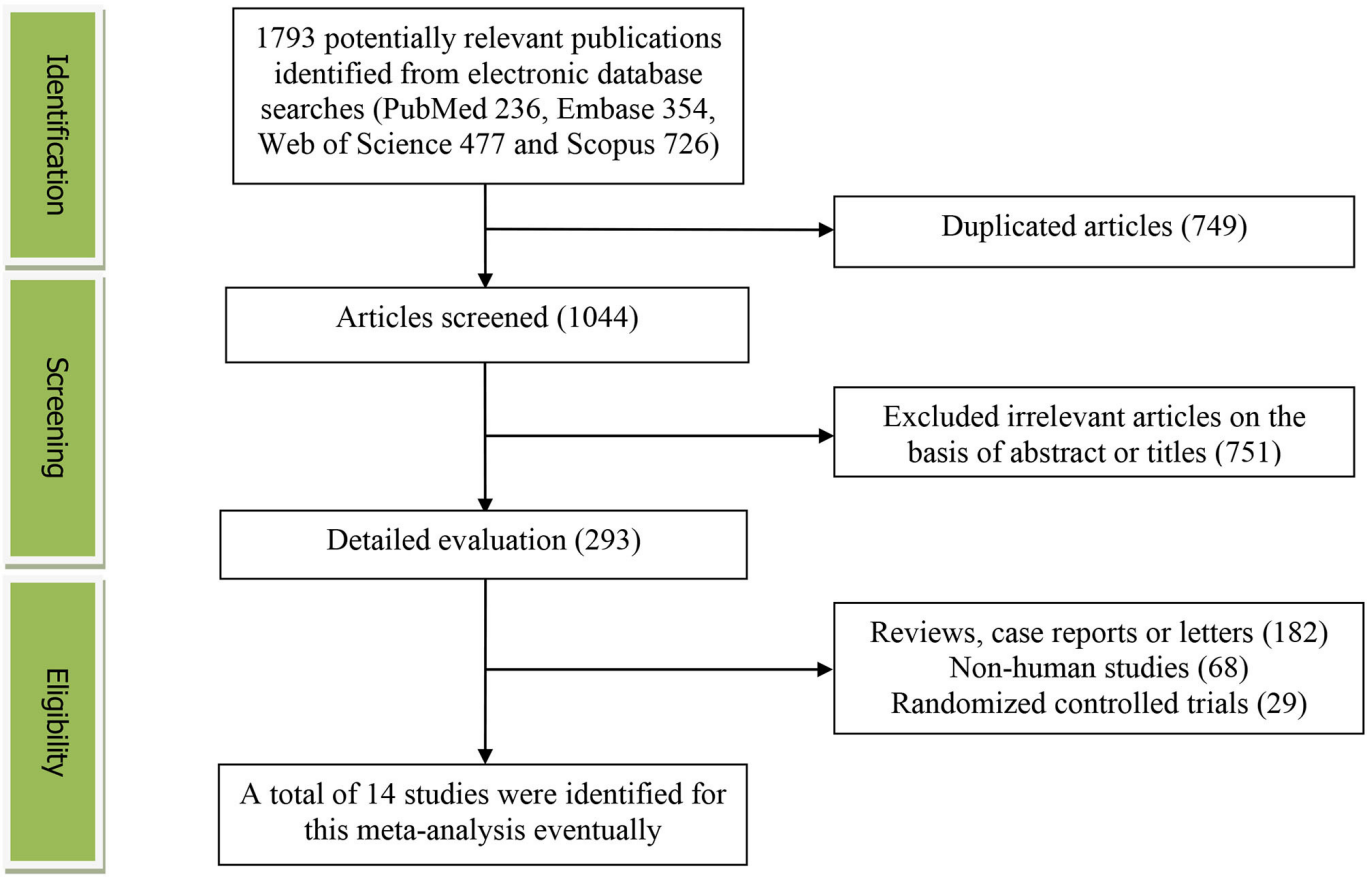
The detailed flow diagram of the study identification and selection in this meta-analysis.

Figure 1 presents the detailed flow diagram of the study identification and selection. A total of 1,793 potentially relevant articles (PubMed: 236, Embase: 354, Web of Science: 477, and Scopus: 726) were retrieved during the initial literature search. After eliminating 749 duplicated articles, 1,044 articles were screened according to the titles and abstracts and 751 irrelevant studies were removed. Then, 182 reviews, case reports, or letters; 68 non-human studies; and 29 randomized controlled trial studies were excluded, respectively. Eventually, a total of 14 studies were identified for this meta-analysis.

### Study Characteristics

Table 1 presents the main characteristics of the included studies. These studies were published between 2010 and 2021. Eight studies were performed in Asian countries [Korea (31), China (32, 35, 37, 39, 41), and Iran (33, 40)]. The other 6 studies were conducted in Brazil (29, 38, 42), USA (30), Spain (36), and Columbia (34), respectively. Except for the study by Bruscato (only female) (29), both the male and female participants were considered. The sample size ranged from 284 to 15,051 for a total of 55,131. The dietary iron, copper, and selenium level were assessed by a food-frequency questionnaire (FFQ) in 3 studies (35, 37, 40) and 24 h or 3-day recall method in 11 studies (29–34, 36, 38, 39, 41, 42). The criteria for MetS were the National Cholesterol Education Program-Adult Treatment Panel III (NCEP ATP III) (31, 32, 38–41), the International Diabetes Federation (IDF) (29, 33, 37), and the American Heart Association (AHA) (30, 35, 36) in 6, 3, and 3 studies, respectively. Moreover, some other criteria (34, 42) were also employed for adolescents.

**Table 1:** Characteristics of the individual studies included in this meta-analysis.

**References**	**Location**	**Age years**	**Gender**	**Sample size**	**Study design**	**Adjustments**	**Exposure**	**Category of exposure**	**Effect estimates**	**Diagnostic criteria of MetS**	**NOS**
Bruscato et al. (29)	Brazil	>60	Female	284	Cross-sectional	Age, smoking, years of education, physical activity and dietary fiber.	24 h recall	Iron		IDF	7
								Quartiles 1	1.00		
								Quartiles 2	0.65 (0.30, 1.38)		
								Quartiles 3	1.33 (0.63, 2.83)		
								Quartiles 4	0.72 (0.30, 1.72)		
Otto et al. (30)	US	45–84	Both	3,828	Cohort	Energy intake, age, sex, race-ethnicity, education, study center, alcohol intake, physical activity, BMI, fiber intake, cigarette smoking, dietary supplement use, the ratio of polyunsaturated fat intake: saturated fat intake and antioxidant intake	24 h recall	Iron Quintiles 1 Quintiles 2 Quintiles 3 Quintiles 4 Quintiles 5	1.00 1.07 (0.85, 1.36) 1.02 (0.80, 1.30) 1.28 (1.01, 1.63) 1.06 (0.81, 1.40)	AHA	8
Choi et al. (31)	Korea	>19	Both	5,136	Cross-sectional	Age, energy intake and alcohol frequency	24 h recall	Copper Male		NCEP ATP III	8
								Quartiles 1	1.00		
								Quartiles 2	0.98 (0.70, 1.37)		
								Quartiles 3	0.85 (0.60, 1.20)		
								Quartiles 4	0.90 (0.60, 1.37)		
								Female			
								Quartiles 1	1.00		
								Quartiles 2	1.07 (0.80, 1.43)		
								Quartiles 3	1.02 (0.74, 1.40)		
								Quartiles 4	0.86 (0.58, 1.27)		
Li et al. (32)	China	18–65	Both	550	Cross-sectional	Age and sex	3 days recall	Copper		NCEP ATP III	7
								Quartiles 1	1.00		
								Quartiles 2	0.75 (0.45, 1.24)		
								Quartiles 3	0.65 (0.39, 1.07)		
								Quartiles 4	0.61 (0.36, 1.01)		
								Selenium			
								Quartiles 1	1.00		
								Quartiles 2	1.38 (0.89, 2.45)		
								Quartiles 3	0.81 (0.54, 1.49)		
								Quartiles 4	0.82 (0.46, 1.30)		
Motamed et al. (33)	Iran	35–65	Both	3,800	Cross-sectional	Sex, age, physical activity level, smoking, past medical history, energy intake and BMI	24 h recall	Iron		IDF	7
								Quintiles 1	1.00		
								Quintiles 2	1.15 (0.90, 1.40)		
								Quintiles 3	1.24 (0.90, 1.50)		
								Quintiles 4	1.24 (1.00, 1.50)		
								Quintiles 5	1.12 (0.90, 1.40)		
								Copper			
								Quintiles 1	1.00		
								Quintiles 2	1.25 (1.00, 1.50)		
								Quintiles 3	1.33 (1.06, 1.60)		
								Quintiles 4	1.16 (0.90, 1.40)		
								Quintiles 5	1.15 (0.90, 1.40)		
								Selenium			
								Quintiles 1	1.00		
								Quintiles 2	0.96 (0.70, 1.10)		
								Quintiles 3	0.85 (0.60, 1.04)		
								Quintiles 4	0.92 (0.70, 1.10)		
								Quintiles 5	0.78 (0.60, 0.90)		
Suarez-Ortegón et al. (34)	Colombia	11–16	Both	1,311	Cross-sectional	Age, BMI, socioeconomic status, and intakes of fat, carbohydrates, protein and ascorbic acid	24 h recall	Copper Males		Ferranti	7
								Tertiles 1	1.00		
								Tertiles 2	/		
								Tertiles 3	0.84 (0.32, 2.19)		
								Females			
								Tertiles 1	1.00		
								Tertiles 2	/		
								Tertiles 3	0.77 (0.30, 1.82)		
Wei et al. (35)	China	>18	Both	2,069	Cross-sectional	Age, sex, cigarette smoking, alcohol drinking, nutritional supplementary, activity level, dietary energy intake, fiber intake and protein intake	FFQ	Selenium Quartiles 1 Quartiles 2 Quartiles 3 Quartiles 4	1.00 0.60 (0.43, 0.86) 0.82 (0.58, 1.17) 0.72 (0.46, 1.14)	AHA	7
Bulka et al. (36)	Spain	18–74	Both	15,051	Cross-sectional	Energy intake, age, gender, and Hispanic/Latino background	24 h recall	Copper Below EAR Normal	1.00 0.81 (0.69, 0.95)	AHA	8
Qu et al. (37)	China	20–75	Both	9,108	Cross-sectional	Age, sex, BMI, physical activity, drinking, and smoking	FFQ	Copper		IDF	8
								Quartiles 1	1.00		
								Quartiles 2	0.95 (0.82, 1.11)		
								Quartiles 3	0.85 (0.74, 0.99)		
								Quartiles 4	0.81 (0.70, 0.94)		
Zhu et al. (39)	China	>18	Both	3,099	Cross-sectional	Age, sex, income, physical activity level, intentional physical exercise, smoking status, alcohol use and dietary total energy intake	24 h and 3 days recall	Iron		NCEP ATP III	8
								Quartiles 1	1.00		
								Quartiles 2	1.37 (1.06, 1.78)		
								Quartiles 3	1.47 (1.11, 1.94)		
								Quartiles 4	1.59 (1.15, 2.20)		
Vieira et al. (38)	Brazil	>18	Both	591	Cross-sectional	Physical activity, gender, alcohol consumption, household per capita income, BMI, high-sensitivity C-reactive protein, age, smoking status, race, total energy intake, misreporting, saturated fat and vitamin C intakes	24 h recall	Iron Quintiles 1 Quintiles 2 Quintiles 3 Quintiles 4 Quintiles 5	1.00 0.83 (0.36, 2.70) 1.34 (0.63, 2.84) 0.52 (0.26, 1.04) 1.14 (0.54, 2.40)	NCEP ATP III	7
Esfandiar et al. (40)	Iran	>18	Both	4,654	Cohort	Age, sex, baseline BMI, educational level, smoking status, total energy intake, fiber, saturated fat, sodium, vitamin C and magnesium intakes	FFQ	Iron Quartiles 1 Quartiles 2 Quartiles 3 Quartiles 4	1.00 0.97 (0.79, 1.19) 1.10 (0.81, 1.49) 2.04 (0.97, 4.28)	NCEP ATP III	7
Zhu et al. (41)	China	>18	Both	5,323	Cross-sectional	Age, sex, region, years of education, physical activity level, intended physical exercises, smoking status, alcohol use and daily energy intake, zinc and magnesium	24 h and 3 days recall	Iron Quartiles 1 Quartiles 2 Quartiles 3 Quartiles 4	1.00 1.35 (1.10, 1.65) 1.47 (1.15, 1.88) 1.60 (1.21, 2.11)	NCEP ATP III	8
Batista et al. (42)	Brazil	14–19	Both	327	Cross-sectional	Sex, age, maternal education, family income, physical activity, and alcohol consumption	24 h recall	Copper		Cooks	7
								Tertiles 1	1.00		
								Tertiles 2	1.49 (0.56, 4.09)		
								Tertiles 3	0.87 (0.28, 2.70)		
								Selenium			
								Tertiles 1	1.00		
								Tertiles 2	2.17 (0.66, 7.36)		
								Tertiles 3	0.81 (0.30, 2.19)		

### The Relative Risk of MetS for the Highest vs. Lowest Dietary Iron Level

**Figure 2:**
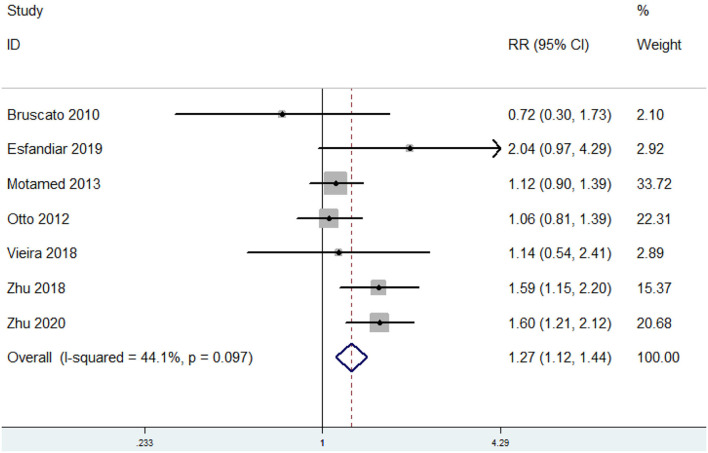
Forest plot of meta-analysis: Overall multi-variable adjusted RR of MetS for the highest vs. lowest dietary iron level.

The overall multivariable adjusted RR showed that the dietary iron level was positively associated with MetS (RR = 1.27, 95% CI: 1.12–1.44; *p* < 0.001) (Figure 2). No substantial level of heterogeneity was obtained among various studies (*p* = 0.097, *I*^2^ = 44.1%). No evidence of publication bias existed according to the Begg's rank correlation test (*p* = 1.000). Table 2 presents the results of subgroup analysis. The above findings were confirmed in cross-sectional (RR = 1.31, 95% CI: 1.13–1.52; *p* < 0.001), Asians (RR = 1.44, 95% CI: 1.13–1.83; *p* = 0.003), the NCEP ATP III (RR = 1.59, 95% CI: 1.30–1.93; *p* < 0.001), >1,000 sample sized (RR = 1.34, 95% CI: 1.09–1.65; *p* = 0.006), 24 h or 3-day recall method (RR = 1.25, 95% CI: 1.10–1.42; *p* < 0.001), and non-heme iron (RR = 1.25, 95% CI: 1.08–1.46; *p* = 0.004) studies, respectively.

**Table 2:** Subgroup analysis of metabolic syndrome (MetS) for the highest vs. lowest dietary iron level category.

**Stratification**	**Number of studies**	**Pooled RR**	**95% CI**	***P-*** **value**	**Heterogeneity**
All studies	7	1.27	1.12, 1.44	*P* < 0.001	*P* = 0.01; *I*^2^ = 44%
**Study design**					
Cross-sectional	5	1.31	1.13, 1.52	*P* < 0.001	*P* = 0.12; *I*^2^ = 45%
Cohort	2	1.34	0.72, 2.47	*P* = 0.35	*P* = 0.10; *I*^2^ = 62%
**Race**					
Asian	4	1.44	1.13, 1.83	*P* = 0.003	*P* = 0.10; *I*^2^ = 53%
American	3	1.04	0.81, 1.32	*P* = 0.77	*P* = 0.69; *I*^2^ = 0%
**Diagnostic criteria of MetS**					
NCEP ATP III	4	1.59	1.30, 1.93	*P* < 0.001	*P* = 0.75; *I*^2^ = 0%
Other	3	1.08	0.91, 1.27	*P* = 0.37	*P* = 0.62; *I*^2^ = 0%
**Sample size**					
<1,000	2	0.94	0.53, 1.66	*P* = 0.83	*P* = 0.43; *I*^2^ = 0%
>1,000	5	1.34	1.09, 1.65	*P* = 0.006	*P* = 0.06; *I*^2^ = 56%
**Exposure assessment**					
FFQ	1	2.04	0.97, 4.29	/	/
24 h or 3 days recall	6	1.25	1.10, 1.42	*P* < 0.001	*P* = 0.10; *I*^2^ = 45%
**Type of iron**					
Heme iron	5	0.98	0.79, 1.22	*P* = 0.88	*P* = 0.04; *I*^2^ = 61%
Non-heme iron	5	1.25	1.08, 1.46	*P* = 0.004	*P* = 0.18; *I*^2^ = 36%

### The Relative Risk of MetS for the Highest vs. Lowest Dietary Copper Level

**Figure 3:**
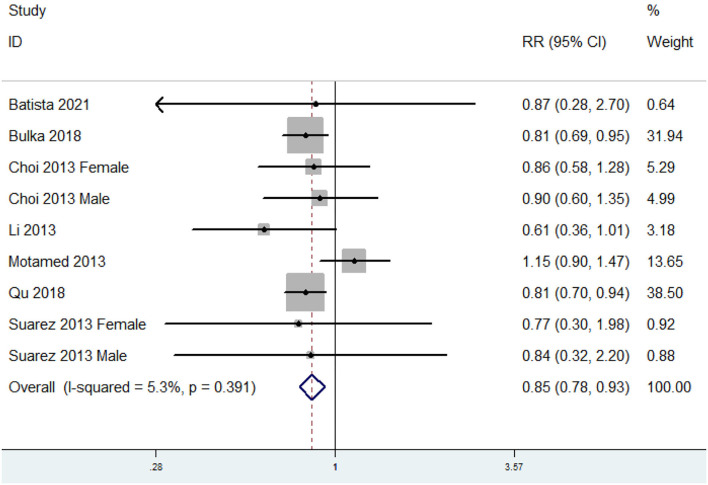
Forest plot of meta-analysis: Overall multi-variable adjusted RR of MetS for the highest vs. lowest dietary copper level.

The overall multivariable adjusted RR showed that the dietary copper level was negatively associated with MetS (RR = 0.85, 95% CI: 0.78–0.93; *p* < 0.001) (Figure 3). No substantial level of heterogeneity was obtained among various studies (*p* = 0.391, *I*^2^ = 5.3%). No evidence of publication bias existed according to the Begg's rank correlation test (*p* = 0.754). Table 3 presents the results of subgroup analysis. The above findings were confirmed in > 1,000 sample sized (RR = 0.86, 95% CI: 0.78–0.94; *p* = 0.001) and adults (RR = 0.85, 95% CI: 0.78–0.93; *p* < 0.001) studies, respectively.

**Table 3:** Subgroup analysis of MetS for the highest vs. lowest dietary copper level category.

**Stratification**	**Number of studies**	**Pooled RR**	**95% CI**	***P-*** **value**	**Heterogeneity**
All studies	7	0.85	0.78, 0.93	*P* < 0.001	*P* = 0.39; *I*^2^ = 5%
**Study design**					
Cross-sectional	7	0.85	0.78, 0.93	*P* < 0.001	*P* = 0.39; *I*^2^ = 5%
Cohort	/	/	/	/	/
**Race**					
Asian	4	0.87	0.78, 0.97	*P* = 0.01	*P* = 0.10; *I*^2^ = 49%
American	3	0.81	0.69, 0.95	*P* = 0.008	*P* = 1.00; *I*^2^ = 0%
**Diagnostic criteria of MetS**					
NCEP ATP III/IDF	4	0.87	0.78, 0.97	*P* = 0.01	*P* = 0.10; *I*^2^ = 49%
Other	3	0.81	0.69, 0.95	*P* = 0.008	*P* = 1.00; *I*^2^ = 0%
**Sample size**					
<1,000	2	0.64	0.40, 1.02	*P* = 0.06	*P* = 0.57; *I*^2^ = 0%
>1,000	5	0.86	0.78, 0.94	*P* = 0.001	*P* = 0.35; *I*^2^ = 10%
**Exposure assessment**					
FFQ	1	0.81	0.70, 0.94	/	/
24 h or 3 days recall	6	0.87	0.78, 0.98	*P* = 0.02	*P* = 0.35; *I*^2^ = 10%
**Population**					
Adults	5	0.85	0.78, 0.93	*P* < 0.001	*P* = 0.14; *I*^2^ = 41%
Adolescents	2	0.82	0.46, 1.46	*P* = 0.50	*P* = 0.99; *I*^2^ = 0%

### The Relative Risk of MetS for the Highest vs. Lowest Dietary Selenium Level

**Figure 4:**
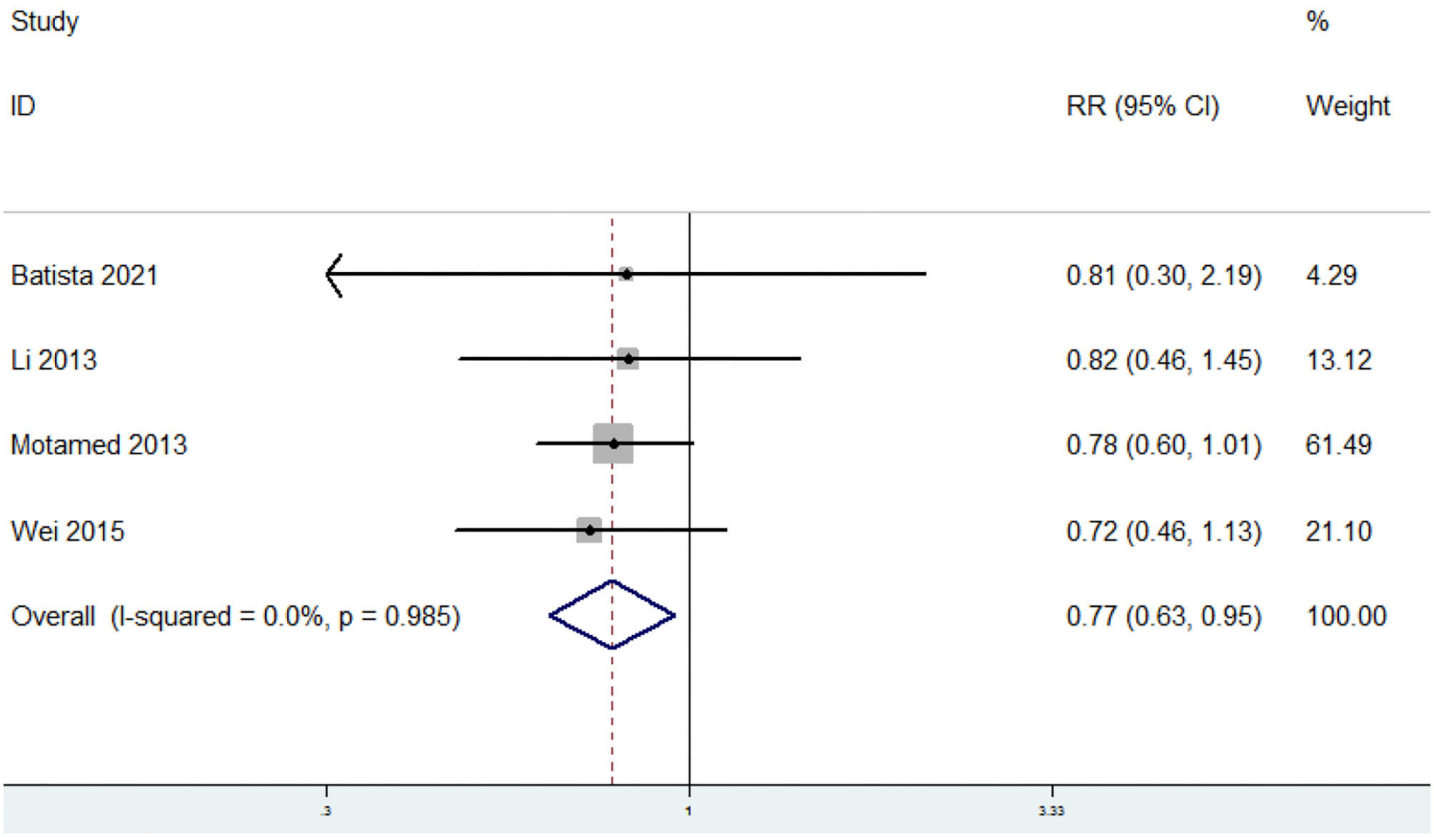
Forest plot of meta-analysis: Overall multi-variable adjusted RR of MetS for the highest vs. lowest dietary selenium level.

The overall multivariable adjusted RR showed that the dietary selenium level was negatively associated with MetS (RR = 0.77, 95% CI: 0.63–0.95; *p* = 0.01) (Figure 4). No substantial level of heterogeneity was obtained among various studies (*p* = 0.985, *I*^2^ = 0.0%). No evidence of publication bias existed according to the Begg's rank correlation test (*p* = 1.000). Table 4 presents the results of subgroup analysis. The above findings were confirmed in Asians (RR = 0.77, 95% CI: 0.63–0.95; *p* = 0.02), the NCEP ATP III/IDF (RR = 0.79, 95% CI: 0.62–1.00; *p* = 0.05), >1,000 sample sized (RR = 0.76, 95% CI: 0.61–0.96; *p* = 0.02), 24 h or 3-day recall (RR = 0.79, 95% CI: 0.62–0.99; *p* = 0.04), and adults (RR = 0.77, 95% CI: 0.63–0.95; *p* = 0.02) studies, respectively.

**Table 4:** Subgroup analysis of MetS for the highest vs. lowest dietary selenium level category.

**Stratification**	**Number of studies**	**Pooled RR**	**95% CI**	***P-*** **value**	**Heterogeneity**
All studies	4	0.77	0.63, 0.95	*P* = 0.01	*P* = 0.99; *I*^2^ = 0%
**Study design**					
Cross-sectional	4	0.77	0.63, 0.95	*P* = 0.01	*P* = 0.99; *I*^2^ = 0%
Cohort	/	/	/	/	/
**Race**					
Asian	3	0.77	0.63, 0.95	*P* = 0.02	*P* = 0.93; *I*^2^ = 0%
American	1	0.81	0.30, 2.19	/	/
**Diagnostic criteria of MetS**					
NCEP ATP III/IDF	2	0.79	0.62, 1.00	*P* = 0.05	*P* = 0.88; *I*^2^ = 0%
Other	2	0.73	0.49, 1.10	*P* = 0.14	*P* = 0.83; *I*^2^ = 0%
**Sample size**					
<1,000	2	0.82	0.50, 1.34	*P* = 0.42	*P* = 0.98; *I*^2^ = 0%
>1,000	2	0.76	0.61, 0.96	*P* = 0.02	*P* = 0.76; *I*^2^ = 0%
**Exposure assessment**					
FFQ	1	0.72	0.46, 1.13	/	/
24 h or 3 days recall	3	0.79	0.62, 0.99	*P* = 0.04	*P* = 0.99; *I*^2^ = 0%
**Population**					
Adults	3	0.77	0.63, 0.95	*P* = 0.02	*P* = 0.93; *I*^2^ = 0%
Adolescents	1	0.81	0.30, 2.19	/	/

## Discussion

A total of 14 observational studies were identified in the present meta-analysis. The pooled results showed that the dietary iron level was positively associated with MetS, whereas a negative association between the dietary copper and selenium level and MetS was obtained.

The pathophysiology of MetS is involved in oxidative stress and inflammation. As a strong pro-oxidant, iron causes oxidative stress and damage to pancreatic beta cells, which decreases the synthesis and secretion of insulin, impairs insulin signaling, and then alters the glucose metabolism (45, 46). Recently, iron-mediated cell death (ferroptosis) has also been reported to induce cardiomyocyte damage and plays an important role in cardiovascular disorders-related pathology (47). On the contrary, copper and selenium are important antioxidants that act against oxidative stress (25, 27). Copper is served as a cofactor of the copper/zinc superoxide dismutase, a protein located in both the cytosol and the mitochondrial inner membrane space to relieve the electron transport chain-generated reactive oxygen species (26). Differently from other metals, selenium works by incorporation into proteins by a cotranslational mechanism (as part of the amino acid selenocysteine) (48). Most selenium proteins participate in antioxidant defense and redox state regulation, particularly the families of glutathione peroxidases and thioredoxin reductases (48). Copper deficiency is associated with the increased high-density lipoprotein (HDL) cholesterol level in rats (49) and blood cholesterol levels in humans (50). Similarly, selenium is also considered to prevent high-fat diet-induced hyperglycemia and liver damage in rats (51) and type 2 diabetes mellitus and cardiovascular disease in humans (52). The above may significantly account for the major findings of this study.

Ferritin, a ubiquitous intracellular protein, is important in the regulation of iron homeostasis (53). Similarly, a meta-analysis of the observational study suggested that the increased ferritin level is positively associated with MetS (54). Moreover, iron chelation therapy (reduce serum ferritin level) was associated with improved serum glucose and HDL levels (55). The phlebotomy with a reduction of body iron could significantly improve cardiovascular risk and glycemic control in patients with MetS (56). The above findings are decent supplements to ours.

Heme iron exists in most animal foods, whereas the rest in animal or plant food is non-heme iron (57). Non-heme iron is absorbed less efficiently than heme iron (~5% non-heme iron and 25% heme iron are absorbed from diet) (41). The epidemiological data indicate that the effect of heme and non-heme iron on MetS may vary from the regional variety (39, 40) and heme iron alone cannot reflect the iron status (58, 59). Indeed, our results with regard to heme and non-heme iron are quite different. The specification of the iron subtype should be considered in further study.

Interestingly, our findings are only confirmed in the NCEP ATP III/IDF diagnostic criteria and > 1,000 sample-sized studies. It is speculated that MetS diagnostic criteria or sample size may influence the results of this study. Moreover, the negative relationship between the dietary iron and selenium and MetS is only specified in the 24 h or 3-day recall method and adult population. Although the number of studies for FFQ and adolescents is limited, the recall method might be precise to reflect the issues and some age-related differences with the dietary pathology of MetS cannot be fully excluded. In addition, the issue of race should also be noted. The corresponding findings only exist in Asians, but not in Americans (with regard to the dietary iron and selenium). Our results suggest that the potential effect of racial variation should not be ignored either.

Our findings can be incorporated into the daily lives of subjects suffering from MetS. The programs to build awareness with collaboration between physicians and nutritionists should be encouraged in the future. For example, reinforce the dietary education in MetS subjects: avoid the dietary iron overdose or copper/selenium deficiency. Nevertheless, the toxicity of excess copper intake should also be emphasized. Excess copper intake is reported to induce oxidative stress, damage to the mitochondrial, contributes to apoptosis, DNA damage, and inflammatory responses (60, 61). Therefore, careful validation by randomized controlled trial/prospective cohort study is still needed before its clinical application.

This study has several strengthens. To begin with, this is the first meta-analysis of observational studies on the associations of the dietary iron, copper, and selenium level with MetS. In addition, the included studies are analyzed based on the adjusted results and large samples. Moreover, the limited heterogeneity level may reflect the decent reliability of our results. Finally, our findings might provide significant information to better consider the dietary effects on MetS.

The limitations of this study should also be acknowledged. First, only 2 prospective cohort studies were identified totally due to the limited relevant literature (causal relationships could not be obtained). Second, the classification of exposure varies greatly among individuals. Third, the adjusted factors and definition of MetS were not uniform. Forth, one included study has combined the data for the dietary iron and red meat as a whole (40). These limitations may weaken the significance of this study.

## Conclusion

Our results suggest that the dietary iron level is positively associated with MetS, whereas a negative association between the dietary copper and selenium level and MetS is obtained. Further large well-designed prospective cohort studies are warranted to elaborate on the issues examined in this study.

## Data Availability Statement

The raw data supporting the conclusions of this article will be made available by the authors, without undue reservation.

## Author Contributions

YZ and JD conceived the idea and drafted this manuscript and selected and retrieved relevant papers. ZL and QL performed the statistical analysis. JL and HG assessed each study. YZ was the guarantor of the overall content. All the authors revised and approved the final version of the manuscript.

## Funding

This study was supported by the National Natural Science Foundation of China (82102581), the National Postdoctoral Science Foundation of China (2021M693562), the Provincial Outstanding Postdoctoral Innovative Talents Program of Hunan (2021RC2020), the Provincial Natural Science Foundation of Hunan (2019JJ40517), the Young Investigator Grant of Xiangya Hospital, Central South University (2020Q14), and the Fuqing Postdoc Program of Xiangya Hospital, Central South University (176).

## Conflict of Interest

The authors declare that the research was conducted in the absence of any commercial or financial relationships that could be construed as a potential conflict of interest.

## Publisher's Note

All claims expressed in this article are solely those of the authors and do not necessarily represent those of their affiliated organizations, or those of the publisher, the editors and the reviewers. Any product that may be evaluated in this article, or claim that may be made by its manufacturer, is not guaranteed or endorsed by the publisher.
